# The Intestinal Microbiome in Early Life: Health and Disease

**DOI:** 10.3389/fimmu.2014.00427

**Published:** 2014-09-05

**Authors:** Marie-Claire Arrieta, Leah T. Stiemsma, Nelly Amenyogbe, Eric M. Brown, Brett Finlay

**Affiliations:** ^1^Michael Smith Laboratories, University of British Columbia, Vancouver, BC, Canada; ^2^Child and Family Research Institute, University of British Columbia, Vancouver, BC, Canada; ^3^Department of Microbiology and Immunology, University of British Columbia, Vancouver, BC, Canada; ^4^Department of Biochemistry and Molecular Biology, University of British Columbia, Vancouver, BC, Canada

**Keywords:** child microbiota, intestinal microbiota, immune-mediated disease, pediatric disease, intestinal dysbiosis

## Abstract

Human microbial colonization begins at birth and continues to develop and modulate in species abundance for about 3 years, until the microbiota becomes adult-like. During the same time period, children experience significant developmental changes that influence their health status as well as their immune system. An ever-expanding number of articles associate several diseases with early-life imbalances of the gut microbiota, also referred to as gut microbial dysbiosis. Whether early-life dysbiosis precedes and plays a role in disease pathogenesis, or simply originates from the disease process itself is a question that is beginning to be answered in a few diseases, including IBD, obesity, and asthma. This review describes the gut microbiome structure and function during the formative first years of life, as well as the environmental factors that determine its composition. It also aims to discuss the recent advances in understanding the role of the early-life gut microbiota in the development of immune-mediated, metabolic, and neurological diseases. A greater understanding of how the early-life gut microbiota impacts our immune development could potentially lead to novel microbial-derived therapies that target disease prevention at an early age.

## Introduction

The infant gut undergoes important developmental stages that are entirely dependent upon the colonization with microorganisms, beginning at birth. Experiments in germ-free animal models have shown that microbial colonization induces anatomical development of the intestinal epithelium into the typical microvilli pattern, increases epithelial cell turnover rates, and kick-starts the maturation of the gut-associated lymphoid (immune) tissue (GALT) (1, 2). Functionally, germ-free mice do not develop oral tolerance (3) and mice treated with antibiotics are far more susceptible to intestinal pathogens (4–6). Furthermore, upon antibiotic treatment, several animal models show either an exacerbated or improved phenotype of immune-mediated diseases, including asthma (7) and type 1 diabetes (8), emphasizing the role of microbiota in the development of immune-mediated diseases.

The behavior of children in the first 3 years of life clearly promotes significant exposure to microbes: feeding directly from maternal skin, constant introduction of hands, feet, and other objects to mouth, and contact of hands onto floor surfaces, especially during crawling and early walking stages. Children also suffer more infectious diseases than adults. Not surprisingly, the microbiota in children under 3 years of age fluctuates substantially and is more impressionable to environmental factors than the adult microbiota (9). Modern changes in lifestyle, including improved sanitization, cesarean sections, antibiotic usage, and immunizations are among some of the factors that can shift the microbiota, and are being studied as potential drivers of the sudden increase in immune-mediated diseases in the developed world. It has been hypothesized that there is a “critical window” early in life during which the microbiota can be disrupted in a way that may favor the development of disease later in life (10). This has been shown to be the case in an animal model of asthma, in which antibiotic treatment exclusively during the perinatal period leads to a more severe disease phenotype (7).

The aim of this review is to summarize the latest findings of childhood microbiota studies focusing on the first 3 years of life. In addition, we compile some of the most relevant prenatal, perinatal, and postnatal events that are known to alter the early-life gut microbiome. We also discuss the increasing evidence indicating a role for microbiota changes during early-life impacting the development of intestinal and extra intestinal diseases. As the study of the microbiota has changed drastically with the emergence of culture-independent techniques, we begin this review by describing the high-throughput sequencing methods currently used to analyze microbial communities.

## Methods to Study the Microbiota

Our concept of the microbiota has profoundly expanded with the emergence of molecular methods to study microbial communities. These methods have eclipsed the small proportion of bacterial groups capable of growing in cultures. Although in other natural environments, the cultivation rates are slightly higher (11, 12), cultivability of human-associated communities remains very low (13, 14). These methods have also led to the discovery of novel taxa, such as the phylum TM7 in the human intestinal tract and other body sites (15–18). It is beyond the scope of this review to discuss all methods to study the microbiota as there are already reviews dedicated to this topic (19–21). Instead, we discuss the most relevant methods used in microbiota research, including targeted approaches like 16S rRNA gene next-generation sequencing (NGS), as well as the large-scale metagenomics approach, also known as shot-gun sequencing.

### 16S rRNA gene NGS – microbiota community surveys

The current gold standard for microbial community analysis is the amplification of the 16S rRNA gene, although other bacteria-specific targets such as the ribosomal 23s subunit and the internally transcribed spacer (ITS) 16S-23S spacer region (22, 23) have been used. The 16s rRNA gene has the advantages of encoding several conserved regions that are exclusive to all bacteria and hypervariable regions that confer specificity to a large number of bacterial species. Current taxonomy reference databases such as SILVA (24) contain over 3 million aligned 16S sequences. The typical 16S community analysis involves DNA or RNA extraction, amplification, standardization, library construction, sequencing, and subsequent bioinformatic analysis. The choice of primers to amplify the desired region of the 16S gene, as well as the extraction protocol used, may introduce bias to the microbial composition results. For example, primers that target regions V1–V2 fail to amplify important bacterial groups, such as *Bifidobacterium*. The use of these primers led to the erroneous conclusion that *Bifidobacteria* were an unimportant bacterial group in the infant intestine (25). Although there is still no consensus on this, comparative analysis with full length Sanger sequencing of the 16S rRNA gene supports the use of primers that amplify the V4 region (26), as they yield the most taxonomically informative sequences (27).

Currently, there are two main sequencing platforms being used by most research groups: the Roche 454 pyrosequencer (GS, FLX, and FLX Titanium) and the Illumina sequencer (MiSeq and HiSeq2000; Illumina Inc., San Diego, CA, USA). In brief, 454 pyrosequencing allows reading DNA fragments of up to 500 bp, allowing the coverage of multiple hypervariable V regions, therefore improving the taxonomic resolution of the dataset. The Illumina platform provides sequencing of DNA fragments of up to 150 bp in length but it sequences 10–100 more samples, at a much higher sequencing depth and lower cost than the 454 pyrosequencer. By choosing the appropriate primers, one can achieve an overlap in the sequences, reducing the sequencing error, and increasing the taxonomic information that can be obtained from the reads. Principal component analysis from a large cohort study that used both 454 and Illumina platforms showed that reads from Illumina clustered very close to the reads obtained from full length Sanger sequencing, whereas reads from 454 pyrosequencing clustered separately (28).

There is not a sequencing technique that is considered gold standard yet, but it is very important to choose the sample preparation protocols and sequencing platforms that will most benefit each study, taking into consideration the environment sampled, the number of samples, cost, and depth of sequencing desired, among other factors.

### Metagenomics

While 16S rRNA gene analysis of the microbial communities gives a survey of the bacteria present in a particular environment, it fails to provide any functional information. For human clinical studies, this means that one cannot infer a possible mechanism that explains the associations between differences in microbial communities and particular disease states. One approach to obtain functional biochemical information is metagenomics. These large-scale studies aim to provide a gene-based inventory of the microbial community. It involves sequencing of sheared genomic DNA without any previous amplification, also known as shot-gun sequencing. To avoid missing the genetic information from very low abundant species, metagenomic sequencing must be done to a very high depth. This was evidenced in the recent Human Microbiome Project study, in which each sample yielded ~10^7^ reads (29). The same NGS platforms discussed for targeted amplicon studies are used in metagenomic sequencing. The short sequences obtained are assembled into longer contiguous reads (contigs) and compared to databases of known genes, such as the NIH Genbank. There are also databases, such as the Kyoto Encyclopedia of Genes and Genomes (KEGG) (30) that organize the genes into biochemical pathways.

The superiority of nucleic acid-based methods to study the microbiota over culture-based methods is very well established. Although methods like metagenomics remain technically sophisticated and expensive to many laboratories, NGS of the 16S rRNA gene is an affordable and a very informative choice.

## Early-Life Gut Microbial Composition

Age is the major driver of differences in gut microbiota in several human studies (31). Using sequential fecal sampling from one infant during the first two and a half years of life, and two large cohort human microbiome studies across North America, Africa, South America, and Europe, it is apparent the gut microbiome is highly unstable during the first 3 years of life (9, 32, 33). Children younger than 3 years of age have a significantly lower diversity index compared to adults, with ~1000 operational taxonomic units (OTUs) detected in the first year of life, compared to almost 2000 OTUs after this. However, while the gut microbiota of infants is dominated by fewer bacterial species, the interindividual variability in this age group is significantly higher than in adults (33).

**Figure 1:**
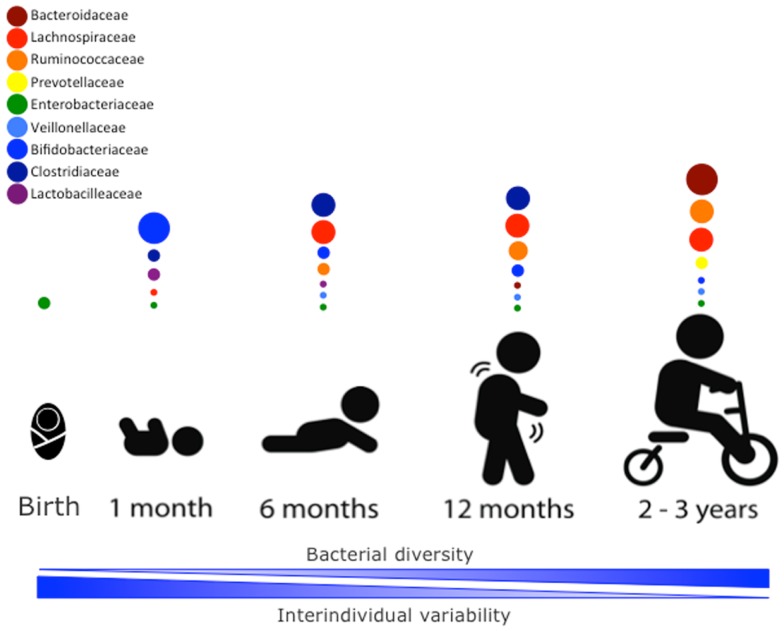
**Stages of microbial colonization of the infant and child intestine**. Most abundant bacterial families are depicted in circles, where the size of the circle is proportional to the relative abundance of the bacterial taxa at each growth stage. The intestinal microbiota of the newborn is initially colonized by *Enterobacteria*. In the days after, strict anaerobic bacteria dominate the microbial community. During the first month, bifidobacterial species predominate in the gut, but the introduction of solid foods at around 4–6 months is accompanied by an expansion of clostridial species (Lachnospiracea, Clostridiaceae, and Ruminococcaceae). Members of the Ruminococcaceae family continue to increase in abundance in the following months. By 2–3 years of age, the microbiota composition consists of mainly Bacteroidaceae, Lachnospiraceae, and Ruminococcaceae, which then remains stable into adulthood.

The newborn intestine at birth is an aerobic environment where only facultative anaerobes, such as members of the Enterobacteriaceae family can grow. In a matter of days, however, the intestinal lumen turns anaerobic, allowing for strict anaerobes, such as *Bifidobacterium, Clostridium*, and *Bacteroides* to colonize (34). During the first few weeks, the microbiota of the newborn gut resembles the maternal skin and vaginal microbiome, with Enterococcaceae, Streptococcaceae, Lactobacillaceae, Clostridiaceae, and Bifidobacteriaceae being predominant bacterial taxa. During the first few months, the diet of the infant is almost exclusively milk, favoring milk oligosaccharide fermenters, such as *Bifidobacterium* to thrive. Many bifidobacterial species have been isolated from the infant gut (35–37) and it is considered the most prevalent bacterial group at this stage (31). Weaning and/or introduction of solids foods mark another rapid and important shift in gut microbiota. The introduction of a variety of nutrients, many of which are polysaccharides not digested by host enzymes, triggers an increase in abundance of *Bacteroides, Clostridium, Ruminococcus*, and a decrease in *Bifidobacterium* and Enterobacteriaceae (9, 38). In the ensuing 12–30 months, the infant gut microbiota progresses into an adult-like gut microbiota abundant in Ruminococcaceae, Lachnospiraceae, Bacteroidaceae, and Prevotellaceae (31) (Figure 1).

Many factors determine the establishment and composition of microbial communities in all mucose, including the gut. The most important microbial inoculum occurs at birth and shortly afterward. However, the type of diet babies and toddlers ingest, their geographical location, and the use of antibiotics during this period of life can have life-long effects on the composition and function of their gastrointestinal microbiota. Birth was once thought to be the first microbial exposure to the infant, but there is now sufficient evidence to support that prenatal microbial exposure occurs. The following section begins by exploring *in utero* encounters with microorganisms.

## Factors That Influence the Development of the Gut Microbiota in Children

### Prenatal exposure

The development of the microbiota begins well before the infant is born. Contrary to what was previously thought, amniotic fluid is not sterile (39, 40). In some cases, bacterial presence in the amniotic fluid is associated with a diseased state. *Mycoplasma* and *Ureaplasma* in the amniotic fluid are frequent isolates associated with health detriments such as chorioamnionitis, pre-term delivery, and necrotizing enterocolitis (NEC) (41–43). Also, women with vaginal infections are much more likely to deliver pre-term (PT) babies (44). Aside from this, bacteria are also often detected in the amniotic fluid and placentas of full-term healthy infants (45–47). Other phyla detected in amniotic fluid and placenta overlap with phyla commonly found in the oral microbiota: Firmicutes, Bacteroidetes, Actinobacteria, Proteobacteria, and Fusobacteria (40, 48). Meconium is also not sterile (49–51), which supports the notion that microbes in the amniotic fluid have access to the unborn fetus. A recent study compared meconium microbiota of pre-term infants to separate datasets of amniotic, vaginal, and oral cavity microbiotas, finding that most overlap between the meconium was from amniotic datasets (52). The bacterial taxa found in meconium using both culture-dependent and independent approaches overlap with adult intestinal microbiota. Enterobacteriaceae (including *Escherichia coli* and *Shigella* spp.), Enterococci, Streptococci, Staphylococci (including *Staphylococcus epidermidis*), and *Bifidobacteria* have been detected in healthy, full-term infants (50, 53). Furthermore, administration of *Enterococcus faecium* to pregnant rats allowed for isolation of the same bacteria from the meconium of term pups immediately after birth by caesarian section (53). Thus, while exposure to pathogenic vaginal microbes may be considered infectious events, prenatal exposure to fecal microbes is likely a natural part of *in utero* development. How these microbes gain access to the uterus remains unknown, although bacterial translocation from the gut into the bloodstream and then to the uterus is one theory that has been proposed, but not tested experimentally yet (54).

### Mode of delivery

Approximately 26% of infants born in Canada are born by cesarean section (55) and this percentage is equal or higher in many other developed countries. The early colonization patterns of cesarean section born infants differ greatly from children born vaginally (34, 56). Knight et al. showed that the first microbiotas of human infants are structured mainly by their mode of delivery, and differences in the bacterial populations within the infant gut are similar to the type of microbiota that the child encounters at birth (31, 56). In fact, post-birth 16S rRNA sequencing data conducted by Knight et al. demonstrate how similar the infant gut microbiota is to the mother’s vaginal or skin microbiota, depending on their mode of birth (56). Additionally, analysis of fecal samples from children 3 days after birth by temporal temperature and denaturing gradient gel electrophoresis (TTGE and DGGE) displayed significant differences in the bacterial populations within the guts of cesarean and vaginally delivered infants (57). Cesarean born infants harbored less *Bifidobacterium* and *Bacteroides* species compared to children born vaginally (57–59). Also, the gut microbiota of cesarean delivered infants at 24 months of age is less diverse than those delivered vaginally (60). The authors hypothesize that this drop in diversity may be due to delayed colonization of the gut by Bacteroidetes, as some C-section delivered infants showed no signs of Bacteroidetes colonization until 1 year of age (60). These differences are not, however, apparent in pre-term infants. Although a shorter gestation time is associated with a higher prevalence of *Clostridium difficile* and *Staphylococcus* species (59), delivery mode has little effect on the development of the premature infant gut microbiota (61). It may also be that the colonization patterns differ in infants born prematurely due to an increase in antibiotics and various medical treatments administered in the neonatal intensive care unit (NICU) (62, 63).

With the establishment of varying gut microbiotas among infants born vaginally or by C-section comes the subsequent development of various immunological disorders associated with mode of birth. Bager et al. show that cesarean birth is associated with a higher risk of development of inflammatory bowel disease (IBD) between 0 and 14 years of age regardless of parental history of IBD (64). Blustein et al. conducted a study including 10,219 children (926 born by C-section) and found that cesarean delivery was consistently associated with adiposity at 6 weeks of age and this association was even stronger if the children were born from obese mothers (65). Additionally, by age 11 these children were 1.83 times more likely to be overweight or obese (65). Furthermore, Decker et al. found that children born by cesarean section also have an enhanced risk for developing celiac disease (66). Gut microbial dysbiosis has been the most accepted explanation for the association of delivery mode with disease outcome, but more research is needed to adequately support these hypotheses, as these remain as association studies.

### Breastfeeding and formula feeding

Human milk satisfies the nutritional requirements of the infant and confers protection against pathogens through the transmission of maternal antibodies (IgA) and other antimicrobial factors (67–72). The World Health Organization (WHO) recommends exclusive breastfeeding of children up to 6 months of age in order to ensure that the growing infant receives the full nutritional benefits of breast-milk (73). A number of studies have compared the effects of breast versus formula feeding (74–76). Human breast-milk harbors its own microbial consortium that is passed on to the infant along with complex non-digestible human milk oligosaccharides (HMOs) that promote the proliferation of specific gut microbes (77). An infant who consumes approximately 800 mL of human breast-milk per day is thought to ingest between 1 × 10^5^ and 1 × 10^7^ commensal bacteria (78), however, the origin of these commensals remains unclear. Bacterial transfer from the mother’s skin during suckling is essentially unavoidable, but a number of studies also support the entero-mammary pathway hypothesis, wherein bacteria from the maternal gut may reach the mammary glands via maternal dendritic cells and macrophages (79). Pyrosequencing of breast-milk from seven mothers revealed the presence of DNA from a number of major gut-associated bacteria (e.g., *Bacteroides* and Clostridia) (80) and it also identified a number of gut-associated bacterial genera shared between the maternal feces, breast-milk, and neonatal feces (79). Furthermore, Martin et al. confirmed that breast-milk and infant feces from mother-infant pairs share the same bacterial strains (81). Regardless of the origin of these gut-associated commensals, a number of studies have attempted to identify the mechanisms by which breastfeeding promotes overall immune health via the entero-mammary pathway (67, 81–84). Comparisons between breast-fed and formula-fed infants show that breast-fed infants tend to contain a more uniform population of gut microbes (84). For example, *Bifidobacteria* and *Lactobacillus* tend to dominate the guts of breast-fed infants whereas formula-fed infants exhibit higher proportions of *Bacteroides, Clostridium, Streptococcus, Enterobacteria*, and *Veillonella* spp. (82–85).

Another area of research regarding formula enrichment is in HMOs and their effects on with the infant gut microbiota. HMOs are considered a type of prebiotic as they promote the growth and proliferation of beneficial commensals and consequently, prevent pathogen colonization of the infant gut and exert positive health effects (86). Certain gut-associated bacterial populations such as *Bifidobacterium* spp. possess gene clusters dedicated to the metabolism of these substrates (87, 88). An *in vitro* study assessed HMO consumption by *Bifidobacterium* spp., *E. coli*, and *Clostridium perfringens* and found that only the *Bifidobacterium* spp. were able to effectively metabolize HMOs (89). Furthermore, the metabolism of these substrates resulted in the production of lactate and short-chain fatty acids (SCFA), which in turn increased the acidity of the surrounding environment, an important factor in preventing pathogen invasion (89). Although *Bifidobacteria* tend to dominate the guts of breast-fed infants, HMOs are consumed by other bacterial taxa and consequently play a large role in the colonization of the infant gut by various microbial species. Unlike *Bifidobacterium* spp., *Bacteroides* spp. (e.g., *Bacteroides fragilis* and *Bacteroides vulgatus*) consume a broad range of HMO glycans (90). A piglet model study investigated the role of formula supplementation with an HMO prebiotic mixture on the gut microbiota and overall health after rotavirus infection (91). The group found that piglets fed the supplemented formula versus those fed formula alone had an increased abundance of butyrate-producing bacteria in the *Lachnospiraceae* family in addition to a reduced duration of rotavirus-induced diarrhea (91). Continued research regarding the role of infant feeding methods in the development of the gut microbiota will likely shed light on the immune and metabolic mechanisms that promote overall infant health.

### Introduction to solid foods

The introduction of solid foods into the infant gastrointestinal tract (GIT) plays a significant role in the development of the early-life gut microbiota. The gut microbiota conducts a much broader range of metabolic processes than the mammalian cells of the GIT, as it produces a number of degradative enzymes that are not encoded by mammalian genomes (92). Consequently, non-digestible carbohydrates such as plant cell wall polysaccharides, cellulose, and xylans can be broken down and fermented by the gut microbiota (93). SCFAs (the most common being butyrate, acetate, and propionate) are end products of microbial fermentation and are essential energy sources for cells in the mammalian gut in addition to being precursors for gluconeogenesis, liponeogenesis, and protein and cholesterol synthesis (94). The types and amounts of SCFAs produced as well as the prevalence of gut microbial species that produce them are determined by the types of carbohydrates consumed (93–95). Additionally, changes in diet can shift the types and prevalence of microbial species in the gut, as certain microbial species may be better equipped to utilize specific substrates (i.e., inulin and fructo-oligosaccharides promote *Bifidobacterium* growth) (93). Conversely, some bacterial phyla (e.g., Bacteroidetes) produce numerous carbohydrate-active enzymes that cover a large spectrum of substrates allowing them to switch between energy sources depending on what is available to them (96).

The first introduction of infants to solid foods occurs during weaning (complimentary feeding) when infants are exposed to a much larger array of non-digestible carbohydrates than those present in breast-milk or formula (38). As discussed above, shifts in diet can significantly alter the gut microbiota due to the presence of new substrates that promote the survival and proliferation of varied types of microbial species (38, 93). Additionally, the pancreatic function, small intestinal absorption, and colonic fermentation abilities of the weaning infant mature with the introduction of non-digestible carbohydrates – changing the overall conditions of the digestive tract and the materials that eventually reach the developing colon (97). Fallani et al. used fluorescent *in situ* hybridization (FISH) to characterize the gut microbiotas of 531 infants across Europe before and after weaning (38). They found that weaning was associated with a significant decrease in proportions of *Bifidobacteria* and *Enterobacteria* species as well as in *C. difficile* and *C. perfringens* (38). Conversely, there was a significant increase in proportions of *Clostridium coccoides* and *Clostridium leptum* (38). In a recent study, 45 exclusively breast-fed 5-month-old infants were randomly assigned to 1 of 3 feeding groups to assess the effect of iron supplementation on the enteric microbiota (98). Children were either fed pureed meats, iron and zinc fortified cereals, or iron-only fortified cereals as the primary complementary food until they were 9–10 months old and fecal samples from 5 to 9 months of age were compared among the children in the three groups (98). Actinobacterial taxa (such as *Bifidobacteria* and *Rothia*) as well as Lactobacillales decreased over time with *Bacteroides* remaining the most abundant in children fed iron-only fortified cereals (98). *Clostridium* group XIVa (large group of butyrate-producing bacteria) was much more abundant in the microbiotas of the children that were fed meat (98). Koenig et al. found that the introduction of formula and peas to the infant’s diet was associated with an increase in the Bacteroidetes phylum and, after metagenomic analysis, an increase in functional adult-microbiome genes associated with carbohydrate utilization and vitamin biosynthesis (9). The introduction of solid foods to the infant diet seems to initiate the maturation of the infant gut microbiota toward that of an adult. However, more research is needed to clarify what specific components of a solid food diet play the biggest role in developing the infant gut microbiota and how these feeding regimens affect the overall health of the infant.

### Geography

Intestinal microbiota differs by geographical location for a number of reasons. Microbial and environmental pressures can alter both the repertoire of bacterial species inhabiting the region and their abundance. Different ethnogeographic populations have distinct genetic backgrounds, regional diets, and cultural practices. Of course, resource-replete regions also have access to better sanitation and healthcare than developing nations. Thus, when studies are designed to assess geographical differences in intestinal communities, any trends are attributable to a large body of differences other than geographical separation. Still, comparisons between several developed and developing regions have provided some insight into which geography-associated variables are the strongest drivers of microbial diversity.

A comparison between continentally distinct populations led to the emergence of distinct types of community structures, driven by composition of the Bacteroidetes phylum (28). Danish, Spanish, Italian, French, Japanese, and American adults were distinguished by the dominant genera in the phylum being either *Prevotella* or *Bacteroides*, or with a less pronounced *Ruminococcus* signature. Interestingly, children do not develop a microbial community signature with either *Bacteroides* or *Prevotella* until after weaning (32). A longitudinal study on Danish infants revealed that a community structure signature was only detectable after 36 months of life, when the Bacteroidetes phylum, undetectable at 9 or 18 months, expanded in abundance (99).

A sequence-based study compared African children living the rural lifestyle in Burkina Faso (BF) to European urban dwellers in Florence, Italy (EU). These juxtaposed populations were selected because BF represents a society closely resembling the ancestral Neolithic lifestyle, accompanied by high-fiber diet of vegetables, grains, and legumes and absence of processed foods. EU children have a typical western diet saturated in sugars and animal fats, accompanied by a greater caloric intake (32). Compared to EU children, BF children are dominated by Bacteroidetes with a reduced Firmicutes population, along with decreased Proteobacteria and reduced Actinobacteria. The Bacteroidetes-Firmicutes balance has been recently hypothesized to reflect climate, in that Firmicutes dominance is associated with colder climates necessitating increased body fat percentage (100). A community structure signature was found in this study as well, with *Prevotella* dominant microbiotas being exclusive to BF and *Bacteroides* exclusive to EU. Besides the differences in microbial species, BF also had raised levels of SCFAs in their stool.

Further studies expanded the developing vs. developed country comparison to include a large cohort of pediatric and adult samples from urban United States and rural villages in Venezuela and Malawi. A *Prevotella* predominant community signature was found in Malawian and Venezuelan microbiotas, whereas *Bacteroides* predominated in the North American samples (33). In addition, there were far fewer differences between Venezuelan and Malawian microbiotas, compared to the samples from USA. Taxa discriminating the two rural communities belong predominantly to the Firmicutes phylum with Enterococci being more common to Venezuelan babies and different distributions of the Clostridia class separating adults. Moreover, metagenomics revealed enrichment in glycan and urease metabolic pathways in Venezuelan and Malawian babies, indicative of enhanced ability to forage nitrogen and glycans as an energy source from breast-milk. This enhanced metabolic efficiency of Venezuelan and Malawian infant microbiotas may be an adaptation to decreased volume of nutrition available to these infants compared to North Americans. The *Prevotella–Bacteroides* split is also observed when comparing children living in a Bangladeshi slum compared to American children living in affluent neighborhoods (101).

Overall, geography is thought to impact the microbiota primarily based on the regional lifestyle. Microbiotas throughout the USA fail to form discrete clusters despite multicultural and multicenter sampling (33, 102, 103). The same could be said for European populations, which are similar to American populations (28, 104). Meanwhile, the microbiotas in developing nations are divergent, but similar between geographically distinct regions with similar dietary habits (32, 33). Importantly, the Prevotella signature is detectable in both Danish (99) and American (33) cohorts, albeit with much more rarity. Thus, the human infant microbiome, irrespective of genetic background, can develop along contrasting trajectories that are dictated by regional lifestyle and diet.

### Antibiotics

Broad-spectrum antibiotics are often prescribed to infants in the Western world in an attempt to protect the developing child from disease (105). In addition to conferring antibiotic resistance in infancy (106), antibiotic over usage can significantly disrupt the overall ecology of the gut microbiota, alter the abundances of resident gut bacteria, and potentially bias the child toward certain diseases (107–109).

Although the gut microbiota is rather resilient to disruptive factors such as antibiotics (110), the ecology of this dense microbial population can be severely altered if exposed to antibiotics too early in its development and/or for long periods of time (111). This ecological disruption combined with the decreased microbial diversity of the infant gut can provide opportunities for enteric pathogens (112–114). *C. difficile* is a common infection associated with the antibiotic disturbed gut microbiota (109). A study including 53 infants between the ages of 0 and 13 months linked the onset of *C. difficile* infections in infancy with alterations in the infant gut microbiota (114).

Antibiotic usage in early-life can also significantly impact the growth of otherwise dominant bacterial phyla in the human gut (111). A study by Fouhy et al. showed that infants exposed to ampicillin and gentamicin shortly after birth tend to harbor higher proportions of Proteobacteria, Actinobacteria, and *Lactobacillus* than the unexposed children for up to 4 weeks after concluding treatment (111). These sorts of effects are even more visible at the genus level, as seen in a study conducted by Tanaka et al. (105). Terminal restriction fragment length polymorphism (TRFLP) analysis of 26 infants (5 of them received oral broad-spectrum antibiotics) showed that the subjects that received either oral or intravenous antibiotics during the first 4 days of life have less gut microbial diversity as well as an attenuation in colonization with *Bifidobacterium* and an increase in colonization with *Enterococcus* (105).

As stated above, antibiotic exposure in early life can render the infant susceptible to numerous diseases later in life (7, 59, 83, 107, 115). Russell et al. showed that vancomycin treatment of ovalbumin (OVA)-challenged mice in early life altered the relative prevalence of microbial populations within the gut microbiota and consequently increased the susceptibility of these mice to asthma (7). A human study associated vancomycin treatment with increased bile acid and glucose metabolism related to the development of obesity (115). Furthermore, antibiotic therapy given to a mouse model of adiposy in early life alters the relative abundances of bacterial populations in the intestine (107). In addition, they showed that this antibiotic therapy in early-life increases colonic short-chain fatty acid levels and increases in adiposy in these mice by altering the regulation of lipid and cholesterol metabolism (107). There is also evidence of antibiotics playing a role in the development of IBD in children (116–118). Shaw et al. found that antibiotic usage during the first year of life was more common in those diagnosed with IBD later in life (116). These results strongly hint at a link between antibiotic usage and disease onset, but research is needed to fully understand the mechanism by which antibiotic-induced microbial dysbiosis influences early-life immune development.

## Role of Gut Microbial Dysbiosis in Pediatric Disease

This section reviews the most relevant scientific evidence associating alterations of the intestinal microbiota and pediatric diseases. A summary of this section in included as Table 1.

**Table 1:** **Intestinal microbial dysbiosis in pediatric diseases**.

Disease	Evidence of dysbiosis	Microorganisms identified
Necrotizing enterocolitis (NEC)	Antibiotics and formula feeding are risk factors for disease development	Overgrowth of Proteobacteria previous to the onset of NEC (119–124)
Inflammatory bowel disease (IBD)	Disease does not occur in germ-free animals Disease occurs in the presence of certain bacterial species in genetically susceptible animal models Intestinal microbiota is disturbed in children with disease	Bacterial diversity was reduced between CD but not for UC (125, 126) Levels of *F. prausnitzii* were increased in CD compared to controls (125, 127) Increase in Proteobacteria for UC and CD and an absence of Verrucomicrobia in UC patients Taxa negatively associated with CD: *Bacteroides, Bifidobacterium, Blautia*, and *F. prausnitzii* (127) Taxa positively associated CD: *Haemophilus sp*., Neiseriaceae, *Fusobacterium, Haemophilus influenzae*, and *E. coli* (127) Veillonellaceae and Pasteurellaceae specifically associated with deep ulceration in UC (127)
Obesity	Transfer of obese mice microbiota into germ-free resulted in weight gain Antibiotics, including tetracycline, glycopeptide, macrolides, and penicillin, induce weight gain in animals Weight gain in children 1-3 years when antibiotics administered before 6 months of age Azithromycin caused weight gain in children and adolescent patients	Reduced Bacteroidetes species in obese individuals (103, 128–130) Increased *Methanobrevibacter smithii* (131) Increased *Lactobacillus* species (129, 132) Increased *Faecalibacterium prausnitzii* in obese children (133)
Atopy and asthma	Mice deficient in the Toll-like receptor (TLR) 4 gene develop a worsened disease Differences in intestinal microbiota of atopic children compared to healthy controls Vancomycin, but not streptomycin, worsened asthma in mice A meta-analysis of 23 studies concluded that infants born via c-section have a 20% increase in risk of developing asthma during childhood	*Mycobacterium vaccae* (134) and *Helicobacter pylori* (135) significantly reduced airway disease in mice Vancomycin treated mice showed a decrease in *Bacteroides* groups and an increase in members of the Lactobacillaceae family (7) *Clostridium* species induced Tregs and resulted in lower IgE titers in a mouse asthma model (136)
Autism-spectrum disorder (ASD)	Altered microbiota in young children with ASD compared to healthy controls Vancomycin ameliorated ASD symptoms in a small group of children	*Bacteroides fragilis* treatment reduced neurological defects in mouse model of ASD (137)

### Necrotizing enterocolitis

One of the most lethal threats to a PT infant is NEC (138, 139). With roughly 7% of low birth weight (LBW) infants being diagnosed with NEC in Canada and USA (140, 141) and up to 30% of these infants going on to die from this disease (142), there is an enormous need to understand its etiology in order to develop novel therapeutic interventions. NEC can have different presentations depending on the age of the newborn and the gestational time. NEC in the term infant is often due to structural abnormalities in the intestine and ischemic injury, and often presents within the first week of life (143–145). NEC in the pre-term infant that occurs in the first day of life is often due to intestinal perforation, and is associated with only minor tissue necrosis and lacks the elevated serum cytokine signature seen in classical NEC (146). Classical NEC, which manifests in pre-term infants 1-week after birth or later, is associated with extensive intestinal tissue necrosis (146), elevated serum proinflammatory cytokines (147–149), and bacteremia and endotoxemia (148, 150). The etiology of classical NEC likely involves the intestinal microbiota. The premature neonate gut barrier is leakier than that of term babies (151, 152), a trend that also holds true in animal models of classical NEC (153–155). Increased intestinal permeability promotes bacterial translocation and may account for the endotoxemia and bacteremia associated with NEC. Other risk factors for NEC include antibiotic use (156, 157) and formula feedings (158, 159), both of which are associated with divergent microbial communities. Breastfeeding favors growth of bacteria beneficial to the host while formula feeding might introduce microbes not normally present in the microbiota. The *Chronobacter* genus is one example of an emerging opportunistic pathogen that can contaminate infant formula and is isolated from NEC patients with higher frequency (160). Thus, the presence of unfavorable bacteria in the leaky pre-term gut is being assessed as another risk factor for NEC.

In general, surveys of the PT infant gut show a microbiota dominated by the Firmicutes and Proteobacteria phyla (119–123, 161). Using culture-based methods, pathogenic bacteria were isolated with more frequency from infants diagnosed with NEC (162) vs. controls, with Coagulase-Negative Staphylococci as a common pathogen. Further, culture-independent case–control comparisons sampled the microbiota within a week of diagnosis and found an outgrowth of Proteobacteria in NEC compared to controls (119, 120).

Several prospective longitudinal studies assessed whether PT infants who would go on to develop NEC followed a distinct trajectory with respect to microbial succession. One such study compared PT infants who did or did not develop NEC to healthy term infants. They found that healthy PT infants began converging to a term-like profile around 6 weeks of age while NEC infants had further outgrowth of Proteobacteria at the expense of Firmicutes, in addition to a reduction of lactose fermenters from the Veillonellaceae family (122). The trend toward a microbiota dominated by Proteobacteria has been observed in further studies of the microbiota in NEC (119–124). Stewart et al. had the unique opportunity to analyze the microbiotas of twins discordant for NEC from 5 days old to over a month in age (162). A sudden outgrowth of *E. coli* the week before diagnosis was found only in the NEC twin, with a subsequent outgrowth of *Klebsiella pneumoniae* shortly following diagnosis, but after commencement of antibiotic therapy. A more comprehensive study prospectively evaluated 18 NEC and 35 control PT infants from birth to hospital discharge and found a Proteobacteria bloom 2 weeks before diagnosis, including *K. pneumoniae, C. perfringens*, and *S. epidermidis* (124). A gram-negative gut dominated by Proteobacteria should therefore be considered a significant risk factor for NEC development. Mechanistically, the innate sensor for bacterial lipopolysaccharide (LPS) toll-like receptor (TLR)-4, is expressed more by the pre-term than term infant gut in both humans (163, 164) and rodent models of pre-term NEC (165). Moreover, in such animal models, pups lacking TLR-4 are protected from NEC (163, 165). These findings suggest that while prematurity may be the seed cause for dysbiosis, the microbiota of all pre-term infants does not follow the same trajectory and for those that Proteobacteria outcompetes other commensals, NEC becomes a much bigger threat.

Introduction of beneficial commensals, or probiotics, has shown some promise in reducing NEC incidence. Of note, feeding of *Bifidobacterium* to rat pups improves gut barrier integrity and diminishes NEC incidence (155). This commensal thrives in breast-milk and is found in the term gut a few days after birth, although it is found less in the pre-term gut (166). This, together with other strains of probiotic bacteria, may provide a means to modulate NEC dysbiosis and accelerate the maturation of the prenatal intestine (167).

### Inflammatory bowel disease

Inflammatory bowel diseases comprises Crohn’s disease (CD) and ulcerative colitis (UC), two disorders of the GIT whose global prevalence continues to climb. Europe and North America have the highest levels of both CD and UC incidence, with over 300 per 100,000 cases for CD and 90–505 per 100,000 cases for UC (168) and 20–30% of diagnoses are made in the pediatric population (169). Host genotype plays a role in IBD, and several genome-wide association studies (GWAS) have uncovered hundreds of risk alleles associated with both UC and CD. The majority of these are genes related to immune homeostasis in the intestine such as MUC19, involved in gut barrier function, CARD9 (mucosal defense), CCL8 and IL8R (innate cell recruitment), IL23R (mucosal T cell responses), and NOD2, an associated gene involved in bacterial sensing (170). However, it is unlikely that IBD is solely a host-mediated disease, as these risk alleles still only count for a small proportion associated with disease.

The gene products of many polymorphisms tied to disease are also involved with host conversations with intestinal microbes. Moreover, the increasing rates of disease incidence far outpace genetic drift in the human population, which suggests that the environment plays a major role in IBD pathogenesis. Several aspects of the environment have been linked to pediatric IBD, including stress, diet, antibiotic use, and prenatal exposure to infections and smoking (171), and all of these environmental exposures have also been associated with aberrant intestinal microbiotas. Animal model studies have shown that the intestinal microbiota plays a critical role in disease development, as IBD does not occur under germ-free conditions (172, 173). Analyses of fecal microbiota consistently reveals less bacterial diversity in UC and CD patients compared to controls, with an increased density of adherent bacteria in biopsy samples. The loss of diversity in CD has been attributed to fewer members of the Firmicutes phylum (174). Within the phylum, *Faecalibacterium* and *Roseburia* are depleted while the Proteobacteria family Enterobacteriaceae increases in abundance (175–177). Some of these findings have used hypothesis-generating approaches to place these associations into causal contexts. For example, the reduction of *Faecalibacterium prausnitzii* in IBD microbiota has been observed extensively in the literature (178). *F. prausnitzii* was then shown to induce human T-regulatory cell differentiation *in vitro* (179) as well as improve gut barrier integrity in a mouse model of colitis (180). Studies such as these, among several others, have provided insight into the dysbiosis of adult IBD and further findings are summarized elsewhere (181, 182). A small collection of studies focus on the pediatric population and their findings are highlighted below.

In a small cohort recruited in Scotland, the microbiota of colonoscopy samples were compared between 25 children with UC or CD and 10 healthy controls. Consistent with the adult literature, diversity was reduced between CD and controls, but this was not true for UC. Also in stark contrast to adult studies, levels of *F. prausnitzii* were increased in CD compared to controls (125). An earlier study of children with severe UC found reduced diversity in patients compared to healthy controls, as well as reduced abundance of Firmicutes and Verrucomicrobia with an increase in Proteobacteria including *E. coli* (183). However, while UC children had not received antibiotics for at least 1 month before sampling, they were on corticosteroid therapy, which could have contributed to these trends.

A more comprehensive, sequence-based study by Papa et al. explored the possibility of using stool microbial signatures as a non-invasive diagnostic tool for pediatric IBD. With a cohort of 23 CD, 43 UC patients, and 24 controls, they were able to discriminate not only IBD from controls but also UC and CD, later applying their algorithm to a dataset of adult IBD biopsy samples with some accuracy (126). Bacterial diversity was reduced in IBD patients and this became more apparent during active disease and less so in remission, with an increase in Proteobacteria (including *Escherichia* and *Shigella*). During remission, there was an increase in *Bifidobacterium* abundance. Unique to UC was the complete absence of Verrucomicrobia, which were present in both CD and controls. Interestingly, UC and CD patients were more distinguishable in remission and more similar during active disease.

All of these studies, while showing small trends, suffer from small sample size, confounding effects of treatment, and for some, relying on stool alone instead of biopsy samples. A recent study done by Gevers et al. addressed these pitfalls, using a cohort of 447 children diagnosed with CD alongside 221 controls. They obtained stool together with both ileal and rectal biopsy samples for 16S analysis alongside shot-gun sequencing on a subset of samples (127). Patients were sampled at diagnosis, thus eliminating pharmaceutical treatment as a confounder. Their findings provided several novel insights. Several taxa were negatively associated with CD, including *Bacteroides, Bifidobacterium, Blautia*, and *F. prausnitzii*. Taxa positively associated CD included *Haemophilus* sp., Neiseriaceae, *Fusobacterium, Haemophilus influenzae*, and *E. coli*. Veillonellaceae and Pasteurellaceae were specifically associated with deep ulceration as determined by endoscopy. Most of these trends were much stronger in the ileal samples and would not have been detected in the stool, where all the same microbes were present but at much lower abundance. Gene content, predicted by the PICRUSt algorithm for ileal microbiota or shot-gun sequenced for stool samples, revealed an enrichment of pathobiont-promoting pathways in IBD. These include enriched benzoate metabolism in the ileum, by-products of which have been associated with dysbiosis and Enterobacteriaceae virulence (184, 185). Stool samples were enriched for glycerophospholipid and LPS metabolism, products of which have been found in increased levels in CD and UC biopsy tissues (184). Meanwhile, stool microbiome genes involved in complex carbohydrate metabolism were diminished and may result in decreased utilization of these substrates in the gut (186).

Taken together, there is now sufficient evidence that pediatric IBD is associated with aberrant microbial communities even before treatment occurs. It is still unclear, however, whether the dysbiosis observed at the time of diagnosis is a cause for the disease or an early manifestation of it. Regardless, as a dysbiotic disease, it has been treated with probiotics in several clinical trials. There is very minimal evidence that probiotics are effective at treating or preventing remission of CD in children. However, in UC, the probiotic preparation known as VSL-3 has proven as an effective therapy for the ongoing disease and remission prevention (187).

### Metabolic disease: Obesity and malnutrition

The combination of obesity and malnutrition has had a substantial impact on human health globally. Obesity and diabetes rates have almost doubled since 1980 worldwide, more than 40 million children under 5 were overweight in 2011 (188) and 170 million children are malnourished (189). Diet and nutrition early in life play an important role in these metabolic disorders. However, the prevalence and severity of obesity and malnutrition cannot be attributed to over-eating or food insecurity alone (190). Early-life dietary intake is a strong driver of one’s composition of intestinal microbes (191). In turn, diet driven alteration of the intestinal microbiota can feed back into host metabolism and immunity, with differing consequences dependent on the composition and metabolic potential of the colonizing microbes (192). Early in life, weight gain and height are important measures of human health, and are surrogate markers for nutritional status. In both malnutrition and obesity, the interrelationship between the microbiota, metabolism, and immunity plays an important role in determining outcomes of severity of diseases seen in malnourished and obese humans, such as environmental enteropathy (EE) in malnourished children and early onset of type 2 diabetes in obese children (193).

#### Obesity

In obesity, seminal early research implicated the composition of the intestinal microbiota as mediators of weight gain, as the ratio between Bacteroidetes and Firmicutes in the gut correlated with the ability of the host to extract energy from their diet (128). Furthermore, in twins discordant for obesity, transfer of their microbiota into germ-free mice was sufficient to account for their variations in weight, independent of genetics (194). The mechanisms that the microbiota use to signal a shift in host metabolism include signaling through TLR5 (195), glucagon-like peptide (GLP)-1/2 (196), and mediating systemic LPS levels (197). Thus, not only microbiota function, but altered immune function is linked to the pathophysiology of obesity. Obesity has an inflammatory component that can account for the development of metabolic disease, reflected by higher levels of circulating inflammatory proteins, increased adipokine secretion by tissues, and dysregulated activation of leukocytes across various tissue sites, including the liver and brain (198).

It is now clear that early-life changes in microbiota composition can alter susceptibility to developing obesity later in life. As discussed previously, mode of delivery at birth can alter the early-life microbial community, where the gut microbiota of children delivered by C-section is more similar to that of the skin microbiota, rather than vaginal microbiota. In a study of obese Brazilian children, subjects born by C-section had a significantly higher risk for obesity as young adults compared to those born by vaginal delivery (199). Similarly, children fed infant formula rather than breast-milk during the first 6 months of life were more than twice as likely to be obese later in life (200). Aside from nutritional intake early in life, many children are exposed to antibiotics throughout their childhood. Sub-therapeutic doses of several classes of antibiotics have widely been used as early-life growth-promoters in the agriculture industry for decades. Mechanisms of this effect were unclear until recently, where a study by Cho et al. showed these sub-therapeutic doses are sufficient to alter the intestinal microbiota, which resulted in enrichment of key microbial genes involved in carbohydrate metabolism to create SCFAs, as well as systemic changes to hepatic lipid and cholesterol metabolism, leading to increased adiposity in young mice (107). Thus, low levels or exposure to antibiotics can induce subtle shifts in the microbial ecosystem, selecting for an environment more conducive for weight gain and energy harvest. Epidemiological studies show early-life antibiotic use and obesity are correlated, as states in the U.S. with the highest obesity rates also have the highest rates of antibiotic use (188). Many studies have shown presence/absence of specific microbes can modulate and program life-long changes in immunity (201), yet future studies must assess in greater detail how these changes could impact metabolic disease progression. By understanding the differing energy harvest and metabolic capabilities of each child’s gut microbiota, we may be able to create microbiota-based interventions to reverse susceptibility to obesity early in life. A recent study showed that treatment of obese mice with *Akkermansia muciniphila* reduced high-fat diet-induced metabolic disorders, including fat-mass gain, metabolic endotoxemia, adipose tissue inflammation, and insulin resistance (202). Colonization with *Akkermansia mucinophila* increased intestinal endocannabinoid levels, controlling inflammation, and also increased the thickness of the inner mucus layer. This treatment required viable *A. mucinophila*, highlighting the importance of active metabolic output of the bacterium. This study and future efforts hold hope that susceptibility to obesity could be controlled early in life through the microbiota and disease outcomes of obesity could also be improved by probiotics.

#### Malnutrition

Function and composition of the intestinal microbiota also plays a key role in the development and severity of malnutrition. By transferring the feces from Malawian twins discordant for severe protein malnutrition into germ-free mice, Smith et al. were able to show a direct role for the microbiota in mediating symptoms of malnutrition (203). Furthermore, germ-free mice fed a Malawian diet and Malawian microbes lost weight quickly, and this effect could not be fully reversed after feeding with a diet of therapeutic food, implying changes in the microbiota can persistently alter host immunity and metabolism. Since microbiota can mediate the impact of malnutrition, early-life fecal–oral microbial exposure during a critical window of growth and development in children can have a lasting impact on the host’s ability to derive nutrients from the diet, resulting in EE (204). While fecal-oral pathogen exposure can significantly alter the early-life gut microbiota and lead to diarrhea and stunting (205), EE presents as a sub-clinical disorder characterized by villous blunting, crypt hyperplasia, increased permeability, chronic inflammation, and malabsorption in the small intestine (204, 206). It is hypothesized that in regions with poor sanitation, children become exposed to increased levels of fecal-associated bacteria that can colonize the small intestine and lead to EE. Nutritional interventions are not sufficient to reverse symptoms of malnutrition in two-thirds of children, suggesting that the microbiota composition in the small intestine may skew the interactions between immunity and metabolism to a scenario where nutrient uptake and microbe-host mutualism are not favored (193). In this way, EE is thought to have a major impact on outcomes of malnutrition early in life, which can lead to persistent loss of gut function, immune function and cognitive ability (207).

Mechanisms explaining how the microbiota may impact EE and malnutrition are yet to be elucidated, although there are many studies implicating a link between early-life diet and environmental exposures altering immunity and the microbiota, which can drive the pathophysiology and diseases related to malnutrition in a paradoxically similar way to how the microbiota could impact obesity early in life. As the efficacy of nutritional recovery after early-life malnutrition remains low, a seminal study by Trehan et al. noted a significant improvement in nutritional recovery and mortality rates post-antibiotic treatment in children with severe-acute malnutrition (208). The authors did not sequence the microbiota in these children to correlate differences in recovery rate from severe-malnourished, although one can speculate that the antibiotic treatment (amoxicillin) shifted the microbiota into a compositional arrangement more favorable for nutrient uptake.

A critical early window exists in a child’s life where its environmental exposures (including diet and microbes) can shift the immune–metabolism–microbiota interactions to pathophysiological states, which lead to alterations in host growth rates, metabolism, and immunity, ultimately resulting in diseases relating to obesity and malnutrition (such as type 2 diabetes and EE, respectively). A recent study suggests the impact of diet and environmental change stresses on the host can be passed on maternally to children, through epigenetic modulation of the DNA by methylation (209). Thus, maternal dietary and microbial exposures are also crucial to the development of the microbiota early in life, as children may inherit genes with differing potential for predisposition for malnutrition or obesity, based on the diet of their mother. Future studies must focus not only on early-life therapeutic interventions to promote improved intestinal health to combat obesity and malnutrition, but also focus on maternal health to achieve a holistic approach to quelling the impact malnutrition and obesity have on society today.

### Asthma and atopy

The atopic diseases (atopic dermatitis, allergic rhinitis, allergic conjunctivitis, anaphylaxis, and asthma) are characterized by IgE-mediated hypersensitivity to an external antigen (210). Strachan’s hygiene hypothesis, proposed in 1989, suggested that exposure of children to infectious agents during infancy will decrease their susceptibility to hyper-inflammatory diseases later in life (211). A recent example of testing this theory was conducted by Ege et al. in Central Europe; they found that children growing up on farms experienced a wide range of microbial exposures and tended to be protected from childhood asthma and other atopic diseases (212).

Significant changes in gut microbiota along with noteworthy changes in immune development and diseases paralleled the industrial revolution, suggesting that environmentally induced changes in the gut microbiota are associated with the development of hypersensitivity diseases. The prevalence of asthma and allergies has continued to rise in industrialized countries and in developing countries where living conditions and hygiene standards are becoming more like those of the Western world (213). Consequently, higher sanitation standards and more readily available antibiotics are likely decreasing our exposure to early childhood microbial antigens at the expense of our immune development (7, 213).

The microflora hypothesis is an extension of Strachan’s hygiene hypothesis in that it argues that there are critical interactions that must occur between our gut microbiota and our immune system in early life in order to circumvent the development of hypersensitivities (214). Studies in germ-free mice show the polarization of the post-birth immune system toward a T-helper 2 cell (T_H_2) driven immune response (215). With the restoration of the gut microbiota, there is a shift toward a T_H_1 and T_H_17 dominated immune phenotype, suggesting that the gut microbiota is important in establishing the balance between the T_H_1/T_H_2 subtypes in early life (a balance often disrupted in subjects with atopy) (215–217).

A number of mouse model studies aim to identify that bacterial taxa play a significant role in preventing or promoting the development of atopy. Perinatal antibiotic treatment of OVA-challenged mice (asthma-induced) has been shown to exacerbate the disease potentially by increasing serum and surface bound IgE as well as decreasing T-regulatory cell accumulation in the colon (218). Arnold et al. show that infection of asthma-induced neonate mice versus adult mice with *Helicobacter pylori* protects these mice from airway hyperresponsiveness, tissue inflammation, and goblet cell metaplasia (common asthma characteristics) (135). Furthermore, Cahenzli et al. provide evidence in mice that a less diverse gut microbiota in early life is associated with elevated mast cell surface bound IgE and exaggerated systemic anaphylaxis (219). Though these studies provide insight for the relationship between gut microbiota and the immune system, researchers are questioning whether specific microbes are actually required to prevent atopy development or whether the metagenomic and metabolomic profiles of the gut microbiota as a whole should be the main focus.

For example, the gut microbiota promotes immune tolerance through the regular stimulation of pattern-recognition receptors (PRRs) and through the production of metabolites (e.g., SCFA) (94, 220). Establishing this immune tolerance to external antigens and host microbes is necessary to prevent the development of hypersensitivity reactions, as T-regulatory cells (+FoxP3) are critical in maintaining the T_H_1/T_H_2 balance (94). SCFAs have been shown to play a role in regulating the proliferation of colonic T-regulatory cells (94). These microbial-derived metabolites may be the key to the microbial-host crosstalk that influences systemic inflammation. Low levels of i-butyric, i-valeric, and valeric acids in stool samples from children 1 year of age were associated with the development of food allergy at 4 years of age; however, analysis of the gut microbiota composition was not conducted (221). A recent study showed that the gut microbiota metabolized a high-fiber diet fed to adult mice and this in turn increased the amount of circulating SCFA and consequently decreased allergic inflammation in the lungs of these mice (95). Additional research that focuses on the early-life microbiota is necessary to determine whether these metabolites may be influencing the development of our immune system and thus potentially biasing us toward or away from certain inflammatory diseases.

The majority of human studies regarding the early-life gut microbiota and the development of atopic disease focuses on the role of variables such as mode of delivery (vaginal vs. cesarean birth), feeding methods (breast-milk vs. formula/solid food diet), and early-life antibiotic exposure. Though the infant gut microbiota is influenced significantly by the child’s mode of birth (56), there are conflicting results regarding the association of birth mode with the development of atopic diseases. For example, according to Kolokotroni et al. children born by cesarean section are at an increased risk of developing asthma later in life, yet van Nimwegen et al. argue that mode of birth is less significant than the place of birth (hospital or home) in the development of the intestinal microbiota and asthma (222, 223).

Breastfeeding is a significant contributor to the development of the infant gut microbiota (224). As discussed earlier in this review, breast-fed babies typically have higher numbers of *Bifidobacteria* and lower numbers of *Bacteroides* and *Atopobium* when compared to infants that are exclusively formula-fed (83, 84), whether or not data such as this can be associated with the protection or promotion of atopic disease is still under debate (225–227). However, higher proportions of certain non-digestible HMOs have been associated with a decreased risk of respiratory disease in infants (228).

Antibiotic treatment during the first few years of life has an equally significant impact on the bacterial ecology of the infant gut (107, 109, 111). Epidemiological studies in humans indicate that broad-spectrum antibiotic exposure may play a role in the development of asthma and atopy. Muc et al. conducted a questionnaire-based study and found that antibiotic exposure in the first year of life plays a significant role in the development of asthma and allergic rhinitis in children (229). Additionally, Hoskin-Parr et al. assessed data from 4952 children enrolled in the Avon Longitudinal Study of Parents and Children and found a dose-dependent association between antibiotic usage during the first 2-years of life and the development of asthma at 7.5 years of age (230).

The development of atopic disease in children is characterized by an extremely complex network of environmental and genetic factors, but as the current research shows, the role of the gut microbiota cannot be ignored.

### Autism-spectrum disorder: The gut-brain axis

The gut–brain axis is the biochemical signaling that takes place between the GIT and the nervous system. This interaction involves the composition and function of the intestinal microbiota, as it has been shown to alter hormones relating to neurochemical changes in the brain that can modulate the behavior of the host, including anxiety, cognition, stress, mood, and energy-level (231). There are many recent reviews covering this phenomenon extensively (232, 233), so for the purposes of this review, we focus on how early-life changes in the microbiota can alter susceptibility to neurological disease, specifically autism-spectrum disorder (ASD). ASD is a collection of neurodevelopmental changes in children where they exhibit complex behavioral changes in their abilities in social interaction and communication, as well as presence of behaviors similar to obsessive–compulsive disorder, including repetitive and narrow interests. While the disease impacts the brain, gastrointestinal symptoms are commonly described in children with ASD (234, 235), and studies have identified the presence of inflammatory infiltrate and histopathology in biopsies of children with ASD, including increased numbers of cytotoxic T cells, CD19^+^ B-cells, and increased enteric IgG1/4 (236, 237). Possibly related to these GIT immune changes and dysfunction, some studies have demonstrated an altered composition of intestinal microbiota in young children with ASD compared to healthy control children with typical neurological function (238–242). These data should be interpreted with caution, however, since these studies are all relatively small and ASD children tend to have alternate diets and antibiotic exposures in relation to healthy, typical children. The microbiota composition may be of clinical importance, as a clinical study treated children with ASD with vancomycin and found a high efficacy of recovery from symptoms (243). However, this was a small study and the validity of antibiotic treatment in ASD needs to be assessed with more data.

When identifying a role for the microbiota in ASD, rather than making predictions based on taxonomic sequence data, recent studies have been studying the metabolite secretions of gut microbes and the impact of these microbiotas on host serum metabolites. A potential mechanism behind ASD symptoms could be neuro-active metabolites mediated by or produced by the microbiota, which could disseminate systemically and penetrate the blood–brain barrier. A recent study by Hsiao et al. sought to understand which bacterially produced metabolites modulate progression of ASD by taking advantage of the maternal immune activation (MIA) mouse model, in which mice are known to display features of ASD. They demonstrated that MIA mice exhibit increased intestinal permeability and alterations in the microbiota and serum metabolite profile compared to control mice (137). Remarkably, the authors found that oral treatment with *B. fragilis* reversed gut permeability and reduced the neurological defects in the MIA mice similar to ASD, including anxiety-like and communicative behavior, while also modulating the serum metabolome. Furthermore, treatment of a single metabolite that is upregulated in MIA mice, restored to normal levels by *B. fragilis*, induced behavioral abnormalities, highlighting the importance of the serum metabolome profile in ASD (137). This research opens up exciting avenues for future studies in ASD and the microbiome in the early stages of life, and hints at the ability of beneficial microbes to modulate symptoms of ASD. Many studies have suggested early-life exposure to *Bifidobacterium* or *Lactobacillus* species modulate behavior in mice (244, 245), and whether these more classical probiotic strains could impact the development of ASD in children remains to be seen. Other studies have altered the hypothesis to argue that it is microbial metabolites meditating the symptoms of ASD. In support of this, elevated SCFA concentrations in the stool of children with ASD have been reported (246). The microbiota early in life produces lower levels of SCFAs compared to adults, since they lack the prevalence of the microbes with the enzymes for their production and generally eat a diet low in complex dietary carbohydrates necessary for the production of SCFAs. Direct exposure of the SCFA propionate to animal brain tissue results in the development of autistic-like behavior (247, 248). Thus, an increase in these metabolites may lead to alterations in brain function.

Once considered in the realms of “pseudo-science,” there has been a groundswell of attention in the role of intestinal microbes and metabolic changes in neurodevelopmental disorders in children, such as ASD. The complexity and heterogeneity of ASD makes it a challenging disease to understand and reverse therapeutically. By taking the new research on the microbiota’s role in the gut-brain access, and the knowledge of importance in the early-life changes, the next-generation of therapies could focus on therapies that increase bacterial homeostasis and barrier function.

## Conclusion

Microbiota surveys worldwide have yielded important lessons regarding what influences the microbial composition of the adult intestine, and how this is associated with different diseases. The shift in the Firmicutes/Bacteroidetes ratio is a constant finding among several of the studies looking at disease. Also, the abundance of *Prevotella* is higher in indigenous regions of the world where there is a much lower incidence of immune-mediated diseases. However, these findings arise from studies done in adults, and the intestinal microbiota during the first 3 years of life differs significantly from adulthood. The microbial community “signatures” associated with the diseases reviewed here do not appear until after 2–3 years, yet it is known that alterations to the early-life microbiota are closely associated the development of these diseases. A recent study in children newly diagnosed with IBD showed that the microbial alterations accompanying disease were different than the changes observed in studies done after diagnosis and during pharmacological treatment (127). This important study suggests that the microbial alterations observed after the disease manifests clinically may not be involved directly in disease pathogenesis and may in fact be, at least in part, a product of the disease manifestations. It is critical to perform additional microbiota studies during the first year of life, a period during which the microbiota composition may give a more accurate portrayal of the microbial conditions that are involved in pathogenesis.

To date, the vast majority of the microbiota studies have focused on the taxonomic profiles of the microbial communities. However, metagenomic studies have shown that different microbes in the gut do not necessarily reflect a different metabolic profile, emphasizing the high degree of redundancy in the biochemical activity among different bacterial taxa (249). Thus, exploring the metagenomic and metabolomic profiles that result from early-life microbial changes associated with disease may provide a better understanding of the role of the microbiota (and their molecules) in the disease process, and may also produce biomarkers of disease before it manifests clinically.

Given that children undergo a drastic succession of microbial changes over a few months, it is clearly important to understand how and when these changes occur. There is a real need for large cohort studies that survey the infant microbiome and metabolome from birth and during at least the first year of life, a period of time most often free of the diseases discussed here yet full of microbial and metabolic changes that may explain the process of later disease emergence. A full understanding of this disease-related changes could allow us to create interventions that rationally shift the microbiota in infants to construct a healthy intestinal environment from a young age. A “critical window” exists early in life where interventions could have more of a profound, long lasting impact on health. Thus, understanding the microbiota changes during this critical window of time will be of great importance for disease prevention.

## Conflict of Interest Statement

The authors declare that the research was conducted in the absence of any commercial or financial relationships that could be construed as a potential conflict of interest.
